# *Emayella augustorita*, New Member of Pasteurellaceae, Isolated from Blood Cultures of Septic Patient

**DOI:** 10.3201/eid3008.231651

**Published:** 2024-08

**Authors:** Sylvain Meyer, Valentin Tilloy, Sylvaine Durand-Fontanier, Thomas Lafon, Fabien Garnier, Christian Martin, Marie-Cécile Ploy, Olivier Barraud

**Affiliations:** Laboratoire de Bactériologie-Virologie-Hygiène, CHU Limoges, Limoges, France (S. Meyer, F. Garnier, C. Martin, M.-C. Ploy, O. Barraud);; Université Limoges, UMR INSERM 1092, Limoges (S. Meyer, F. Garnier, C. Martin, M.-C. Ploy, O. Barraud);; Centre National de Référence des Herpèsvirus, Limoges (V. Tilloy);; Service de Chirurgie Digestive, CHU Limoges, Limoges (S. Durand-Fontanier);; Service d’Accueil des Urgences, CHU Limoges, Limoges (T. Lafon).

**Keywords:** Pasteurellaceae, bacteria, bacteremia, new genus, new species, blood culture, Emayella augustorita, France

## Abstract

We report discovery of a new bacterial genus and species of the family Pasteurellaceae by using phylogenetic and metabolic analysis. The bacterium, *Emayella augustorita*, was isolated from blood cultures of a patient in France diagnosed with an adenocarcinoma of the intestines and who was treated with a biliary prosthesis placement.

Pasteurellaceae bacteria have been identified in many vertebrates as members of the microbiota but can occasionally cause human infections (*1*). Currently, the family Pasteurellaceae contains 34 genera and 105 species (https://pasteurellaceae.eu). Few acquired antibiotic resistances are reported (occasionally β-lactamases, macrolides, tetracyclines, or fluoroquinolones), but recently some strains were reported to have multidrug resistance profiles (*2*–*4*). We describe a new bacterium belonging to the Pasteurellaceae family isolated from positive blood cultures of a septic patient.

A 74-year-old woman was admitted January 2022 to the emergency department at the Limoges teaching hospital in France. She reported complaints of an occlusive syndrome with nausea, vomiting, and fever. She was previously diagnosed in 2015 with an adenocarcinoma of the small intestine with metastases in the lungs and liver with biliary compression. After multiple chemotherapies and surgeries, she was diagnosed with severe bacteremia of digestive origin (Figure). A metallic biliary prosthesis through a biliary drain was placed in December 2021. No documentation of any contact with farm animals or pets was reported.

**Figure:**
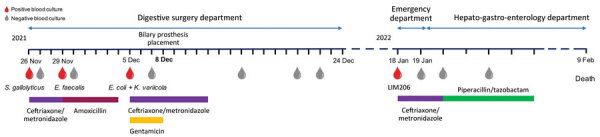
Timeline of main events for case report on the isolation of *Emayella augustorita*, a novel bacterium of the *Pasteurellaceae* family recovered from a patient with sepsis, France. *E. coli*, *Escherichia coli*; *E. faecalis*, *Enterococcus faecalis*; *K. variicola*, *Klebsiella variicola*; *S. gallolyticus*, *Streptococcus* *gallolyticus*.

We conducted an abdominal computed tomography scan that detected a hepatic lesion with a heterogeneous hypodense area of the tip of the VI segment of the liver on the path of the biliary drain, suggesting a biloma. Laboratory results showed evidence of possible infection with a total leukocyte count of 24.4 × 10^9^ leukocytes/L (reference range 3.78–9.42 × 10^9^ leukocytes/L) and a C-reactive protein result of 150 mg/L (reference range <5 mg/L). Two blood cultures were collected, and we initiated empiric antibiotic therapy with ceftriaxone-metronidazole (Figure). Four blood culture bottles, aerobic and anaerobic incubation, detected growth after 16 hours of incubation. A Gram stain of the positive bottles showed a short gram-negative rod (Appendix Figure 1). Small, bright colonies grew in a 5% CO_2_ atmosphere on blood agar and PolyVitex plates (bioMérieux, https://www.biomerieux.com) after 24 hours of incubation at 35°C (Appendix Figure 1). We attempted bacterial identification by using matrix-assisted laser desorption/ionization time-of-flight mass spectrometry (bioMérieux) and did not match any known bacterial species. We determined MICs by using E-tests (bioMérieux) on Mueller Hinton agar with 5% horse blood (bioMérieux) for amoxicillin/clavulanic acid, piperacillin/tazobactam, cefotaxime, levofloxacin, and trimethoprim/sulfamethoxazole, according to pharmacokinetic-pharmacodynamic EUCAST breakpoints (https://www.eucast.org). No phenotypic resistance was detected (Table). The patient’s successive blood cultures became negative, but her general status worsened, leading to death in February 2022 (Figure).

**Table:** MIC values observed for LIM206, *Emayella augustorita*, a novel bacteria of the Pasteurellaceae family recovered from a patient with sepsis, France*

Antibiotic	PK/PD breakpoints, mg/L†	MIC, mg/L
Amoxicillin	2–8	0.75
Amoxicillin + clavulanic acid	2–8	0.75
Piperacillin + tazobactam	8–16	1.0
Cefotaxime	1–2	0.032
Levofloxacin	0.5–1	0.064
Trimethoprim/sulfamethoxazole	0.5	0.004

*PK/PD, pharmacokinetics/pharmacodynamics. †All breakpoints were from EUCAST except for trimethoprim/sulfamethoxazole, which was epidemiologic cutoff value.

Sanger sequencing of the whole 16S rRNA gene (Genbank accession no. OR046993) did not show sufficient identification (94.78% similarity with *Pasteurella oralis*). A 16S phylogenetic tree confirmed the result but also emphasized the wrong affiliation of genera in this family (Appendix Figure 2). Because of the low identity percentage (<97%), we conducted whole-genome sequencing by using the Ion GeneStudio S5 Plus platform (ThermoFisher Scientific, https://www.fishersci.com), as previously described (*5*). The genome size was 2.68 Mbp, and the total DNA guanine and cytosine mol% content was 45.3 mol%. Annotation identified 2462 coding sequence, 45 tRNA, and 6 rRNA. No resistance genes were detected (Appendix). We identified the type strain as LIM206 (Genbank accession no. JAWHQP010000000).

A phylogenetic tree based on single nucleotide polymorphisms comparison between whole genomes of different Pasteurellaceae species showed LIM206 was placed on a separate branch from all other genera. The average nucleotide identity between LIM206 and the closest members that shared a phylogenetic branch was 73.48% with *Actinobacillus succinogenes*, 74.09% with *Basfia succiniciproducens*, 72.13% with *Lonepinella koalarum*, 73.20% with *Mesocricetibacter intestinalis*, and 72.13% with *Pasteurella bettyae* (Appendix Figure 3). Results of those combined analyses suggested LIM206 belonged to a new species and a new genus of Pasteurellaceae. We conducted multilocus sequence analysis on 16S rRNA, *infB*, *recN*, *rpoA*, and *rpoB* genes according to previous recommendations (*6*,*7*) (Appendix). LIM206 was separated from existing genera of Pasteurellaceae and closely linked to *A. succinogenes* and *B. succiniciproducens* (Appendix Figure 4). To confirm the new genus, we conducted amino acid identity analysis and found a maximum amino acid identity value of 77.67% with *Basfia succiniciproducens* (Appendix Figure 5). This identity value was considered below the genus identity threshold of ≈83% compared to other Pasteurellaceae genera. 

We compared the biochemical characteristics of LIM206 to those of different species of the Pasteurellaceae family (Appendix Table 1). We detected the presence of urease activity, the acidification of l-arabinose and d-xylose, and the absence of acidification of d-mannitol and d-trehalose, which are not frequently observed in Pasteurellaceae. Those characteristics are absent in the genetically closest species (*8*,*9*).

In conclusion, we report the description of a new genus and species of the Pasteurellaceae family found in blood cultures of a septic patient in France followed for metastatic adenocarcinoma of the intestines. We derived the genus name *Emayella* from the word enamel; the species name *augustorita* is in reference to the Roman name of Limoges. The bacterium is a short gram-negative coccoid to rodshape. It is catalase negative, oxidase positive, nonmotile, fermentative, capnophilic, and nonhemolytic. The bacterium does not require β-nicotinamide adenine dinucleotide or hemin-factors for growth. 

Appendix Additional information about *Emayella augustorita*, new member of Pasteurellaceae, isolated from blood cultures of septic patient.
