# Supplementary material for: Emayella augustorita, New Member of Pasteurellaceae, Isolated from Blood Cultures of Septic Patient
Source: Emerg Infect Dis. 2024 Aug;30(8):1719–21. doi: 10.3201/eid3008.231651 (PMC11286039; doi:10.3201/eid3008.231651)
Supplement: Appendix — Additional information about Emayella augustorita, new member of Pasteurellaceae, isolated from blood cultures of septic patient. [file 23-1651-Techapp-s1.pdf]

Article DOI: https://doi.org/10.3201/eid3008.231651

EID cannot ensure accessibility for supplementary materials supplied by authors. Readers who have difficulty accessing supplementary content should contact the authors for assistance.

Emayella augustorita, New Member of Pasteurellaceae, Isolated from Blood Cultures of Septic Patient

Appendix

Sanger sequencing of the whole 16S rRNA gene was performed using previously published primers (*1*) and sequences were compared on the EzBioCloud database 2021.07.07 (*2*). Construction of the Neighbor-joining 16S phylogenetic tree was performed on Geneious® Prime 2020.0 software using Tamura-Nei genetic distance model with 1000 bootstraps.

After WGS, a total number of 4,197,739 reads of 219 bp mean length were generated. Reads were assembled with SPADES v3.15.4. yielding 88 contigs above 500 bp. Annotation of the genome was performed with Prokka v1.14.6. Resistance genes were detected from whole genome sequences on CARD v3.2.5 database with standard parameters (*3*).

Single nucleotide polymorphisms (SNP) comparison between whole genomes of different *Pasteurellaceae* species was constructed with snippy v4.4.3 (https://github.com/tseemann/snippy) and Fasttree v2.1.10 programs. Average Nucleotide Identity (ANI) scores were obtained on EZBiocloud platform. Considering Multi Locus Sequence Analysis (MLSA), 16S rRNA, *infB, recN, rpoA* and *rpoB* genes of each genome were merged, aligned using MUSCLE on Geneious® Prime 2020.0 and a Neighbor-joining tree was constructed after 1000 bootstrap replications on MEGA 11 software. Amino-acid identity (AAI) analysis was performed using the EzAAI v1.2.3 pipeline (https://github.com/endixk/ezaai) with the parameters of 40% amino acid identity and 50% coverage length, as recommended by Nicholson et al. (*4*)

Biochemical characteristics were obtained using API20E gallery, Vitek2® NH and Vitek2® ANC cards (bioMérieux).

Table. Phenotypic and biochemical characteristics of LIM206 *Emayella* gen. nov. from other genera of the family Pasteurellaceae.

Genera	1	2	3	4	5	6	7	9	10	11	12	13	14	15	16	17	18	19	21	22	23	24	25	26	27	28	29	31	32	33	34
X and/or V dependency	−/−	-/D	-/D	-/D	−/−	−/−	−/−	−/−	−/−	−/−	−/−	−/−	−/−	-/+	+/+	−/−	−/−	−/−	−/−	−/−	−/−	−/−	−/−	−/−	−/−	−/−	−/−	−/−	−/−	-/+	−/−
β-haemolysis	−	−	−	−	−	D	−	−	+	−	−	−	+	−	D	D	−	D	−	−	−	−	−	−	−	−	+	−	−	+	D
Oxydase	+	−	D	+	+	D	+	+	+	+	+	+	D	+	+	+	−	D	+	+	+	+	+	D	+	+	+	+	+	+	D
Catalase	−	D	D	D	−	D	+	−	+	−	−	+	D	+	D	−	−	+	−	+	+	+	+	+	−	+	+	+	+	+	D
Urease	+	−	+	−	−	−	−	−	−	−	−	−	D	−	D	−	−	-	+	D	−	+	D	D	−	+	−	−	−	−	−
Indole	−	−	−	−	−	−	−	−	−	D	+	+	D	−	D	+	+	-	+	−	−	−	+	+	−	D	−	+	ND	−	−
Alkaline phosphatase	+	+	+	D	+	+	+	+	+	−	+	+	+	+	+	+	-	+	+	−	+	D	+	+	+	+	+	−	−	+	+
PNPG (α-glucosidase)	−	ND	D	D	−	D	−	−	−	+	−	+	D	ND	−	ND	ND	ND	−	+	+	ND	ND	D	ND	+	ND	−	+	D	−
Ornithine decarboxylase (ODC)	−	−	−	D	−	−	−	−	−	+	−	−	−	ND	D	−	−	D	−	−	−	−	−	D	−	D	ND	+	−	−	−
Acid produced from:																															
L-Arabinose	+	−	D	D	−	−	−	+	+	+	+	ND	D	−	ND	ND	+	D	−	−	−	−	+	D	ND	D	+	+	+	+	D
D-Galactose	+	D	D	D	+	−	+	+	ND	+	ND	ND	+	+	+	−	ND	+	ND	+	+	−	ND	+	ND	+	−	ND	ND	ND	+
D-Mannitol	−	D	+	D	+	+	D	−	+	+	+	−	+	−	−	ND	−	+	+	D	−	−	−	D	−	−	−	+	+	D	−
D-Mannose	+	D	D	+	+	+	+	+	+	+	+	+	+	+	D	ND	+	-	+	+	+	−	−	+	−	+	+	ND	+	−	+
L-Rhamnose	−	ND	−	−	−	−	D	−	ND	ND	+	ND	−	−	−	ND	ND	D	D	ND	+	−	−	−	−	−	−	+	+	+	−
Sucrose	+	D	+	+	+	+	+	+	+	+	+	+	+	+	D	−	D	+	+	+	+	−	−	+	−	+	−	+	−	−	+
D-Tréhalose	−	D	D	D	+	+	+	-	+	+	−	D	D	−	−	−	ND	−	−	+	+	−	+	D	−	+	−	+	+	D	−
D-Xylose	+	D	+	−	+	−	−	−	+	−	ND	ND	+	−	D	−	ND	+	ND	D	+	−	−	−	−	+	−	ND	ND	+	−

Genera: 1. LIM206 (data from this study); 2. *Aggregatibacter*; 3. *Actinobacillus sensu stricto*; 4. *Avibacterium*; 5. *Basfia*; 6. *Bibersteinia*; 7. *Bisgaardia;* 8. *Caviibacterium*; 9. *Chelonobacter*; 10. *Conservatibacter*; 11. *Cricetibacter*; 12. *Frederiksenia*; 13. *Gallibacterium*; 14. *Glaesserella*; 15. *Haemophilus sensu stricto*; 16. *Histophilus*; 17. *Lonepinella*; 18. *Mannheimia*; 19. *Mesocricetibacter*; 20. *Muribacter* 21. *Necropsobacter*; 22. *Nicoletella*; 23. *Otariodibacter*; 24. *Pasteurella sensu stricto*; 25. *Phocoenobacter*; 26. *Rodentibacter*; 27. *Seminibacterium*; 28. *Testudinibacter*; 29. *Ursidibacter*; 30. *Vespertiliibacter*; 31. *Volucribacter*; +, positive reaction; -, negative reaction; D, variable reaction; ND, not defined

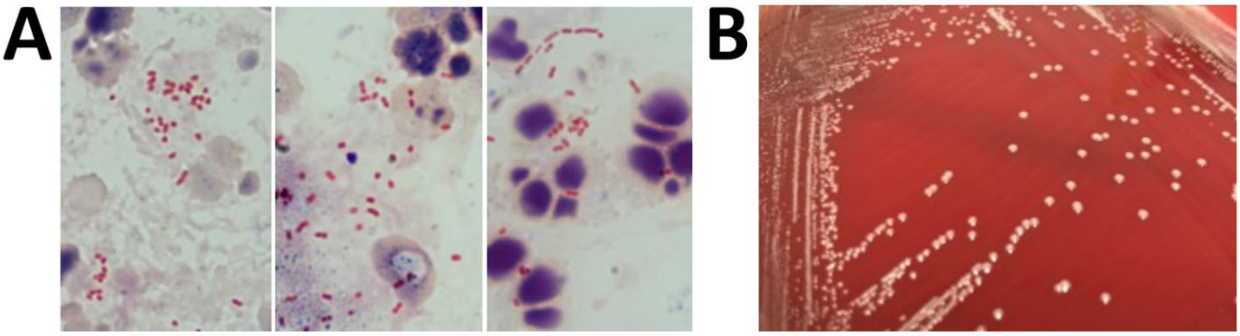

Appendix Figure 1. Gram staining and culture on blood agar of LIM206.

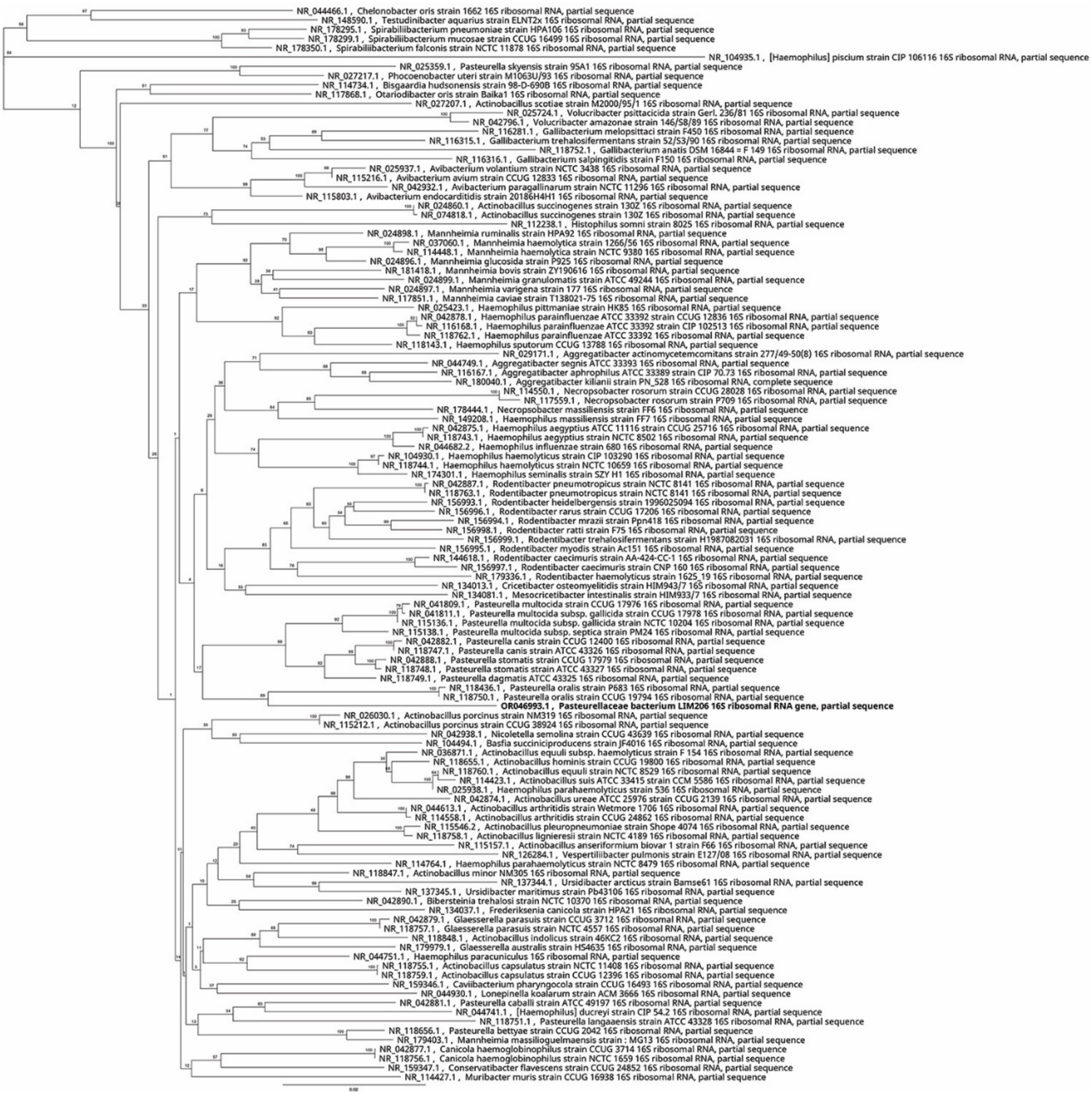

Appendix Figure 2. Neighbor-joining phylogenetic tree based on full 16SrRNA sequences of LIM206 compared to other *Pasteurellaceae* species. This phylogenetic tree has been generated using Geneious® Prime 2020.0 software with Tamura-Nei genetic distance model with 1000 bootstraps. In bold is represented the position of LIM206. Scale bar represents the phylogenetic distances based on 16S sequence differences.

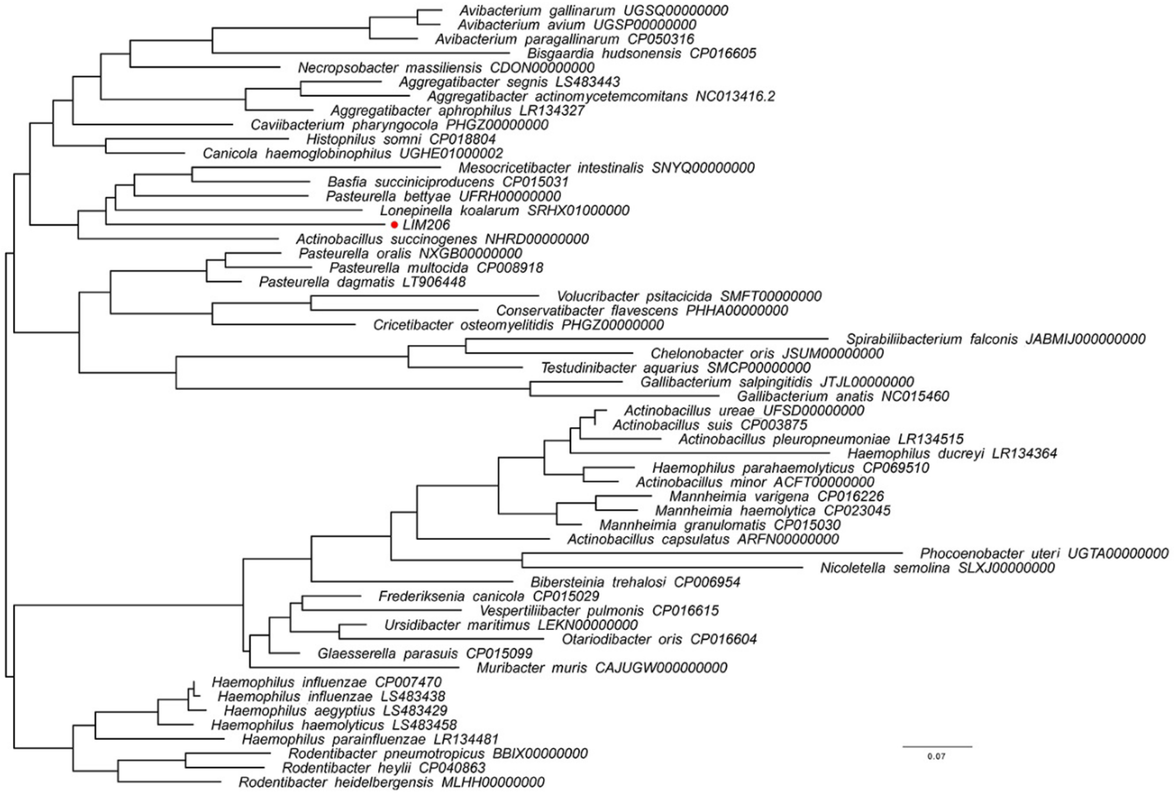

Appendix Figure 3. Maximum likelihood phylogenetic tree based on whole genome sequencing SNP comparison of different *Pasteurellaceae* genomes. SNP differences were generated using snippy v4.4.3. Fasttree v2.1.10 was then used to generate phylogenetic tree with standard parameters. Visualization of the tree was executed on RStudio v2023.9.1. Scale bar represents the distance of nucleotide substitutions per site. Redpoint shows the position of LIM206.

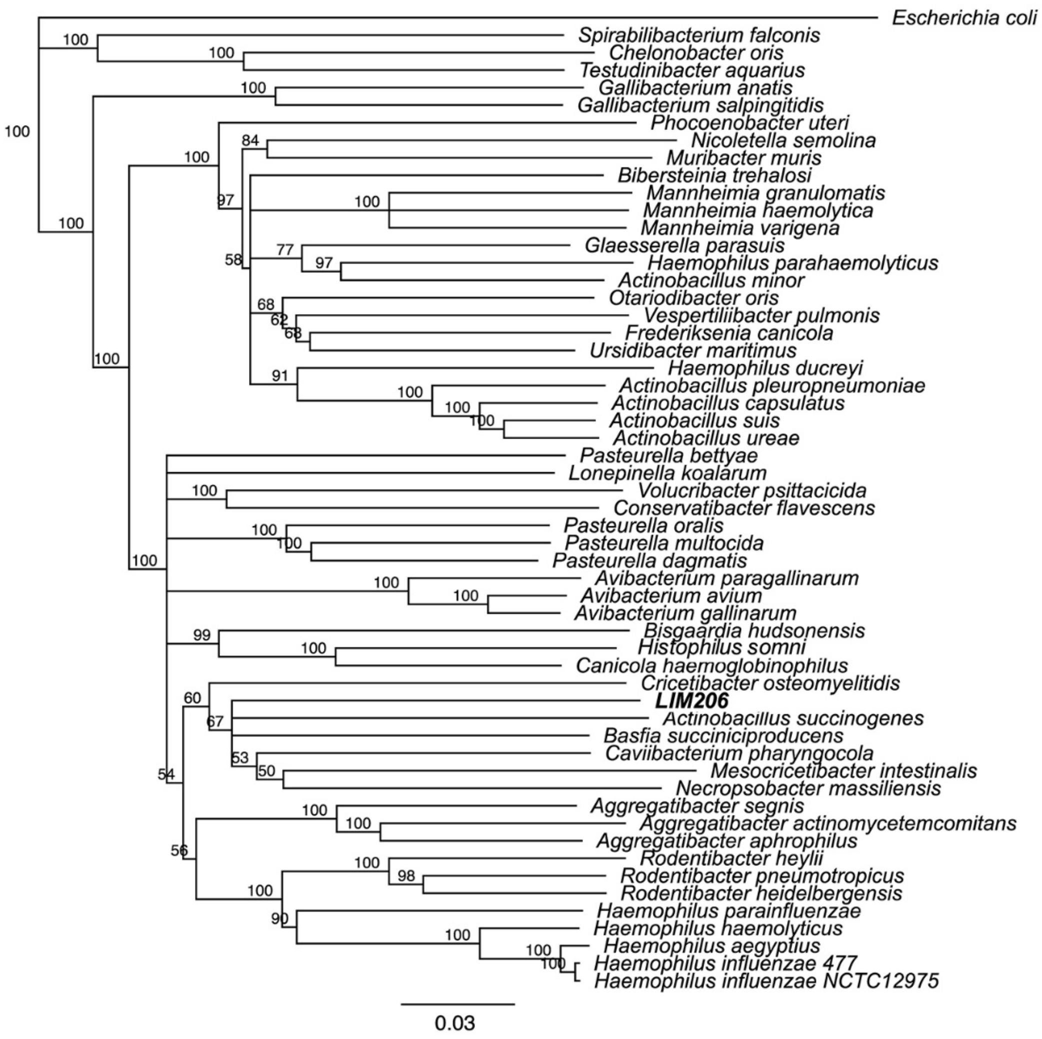

Appendix Figure 4. Neighbor-joining tree based on MLSA of *16SrRNA*, *infB, recN, rpoA* and *rpoB* genes alignment of 54 *Pasteurellaceae* genomes after 1000 boostraps replications**.** Sequences of strain *E. coli* MG1655 (NC_000913.3) were added to root the tree. In bold is represented the position of LIM206.

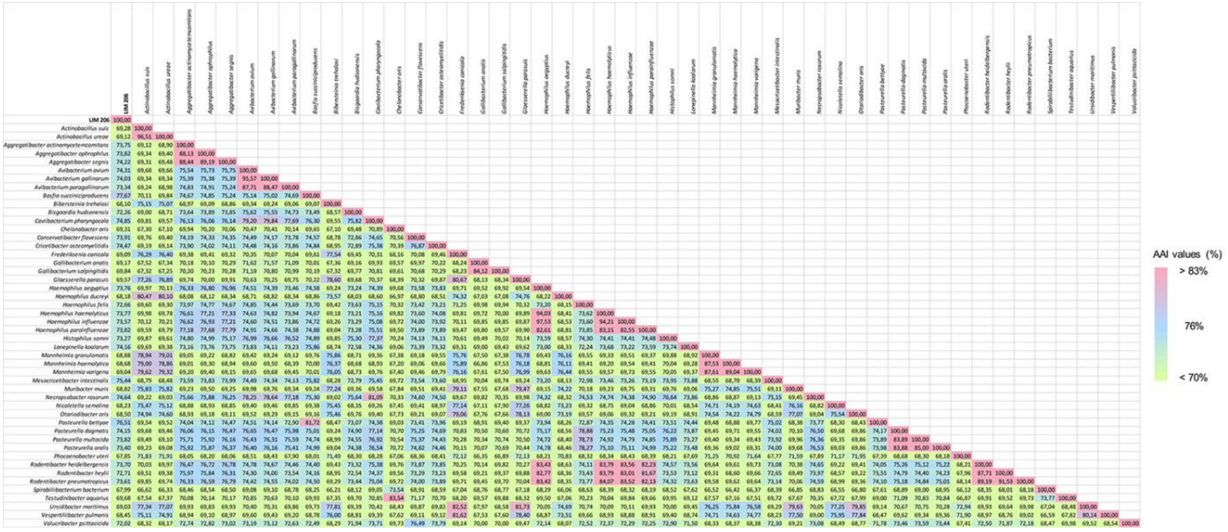

Appendix Figure 5. Matrix of amino-acid identity scores (in %) obtained after comparing main species of the *Pasteurellaceae* family. Analysis was performed on EzAAI v1.2.3 pipeline with standard parameters (40% amino acid identity and 50% coverage length).

References

1. Weisburg WG, Barns SM, Pelletier DA, Lane DJ. 16S ribosomal DNA amplification for phylogenetic study. J Bacteriol. 1991;173:697–703. PubMed https://doi.org/10.1128/jb.173.2.697-703.1991

2. Yoon S-H, Ha S-M, Kwon S, Lim J, Kim Y, Seo H, et al. Introducing EzBioCloud: a taxonomically united database of 16S rRNA gene sequences and whole-genome assemblies. Int J Syst Evol Microbiol. 2017;67(5):1613-1617. **PMID: 28005526**

3. Alcock BP, Raphenya AR, Lau TTY, Tsang KK, Bouchard M, Edalatmand A, et al. CARD 2020: antibiotic resistome surveillance with the comprehensive antibiotic resistance database. Nucleic Acids Res. 2020 8;48(D1):D517-D525. **PMID: 31665441**

4. Nicholson AC, Gulvik CA, Whitney AM, Humrighouse BW, Bell ME, Holmes B, et al. Division of the genus *Chryseobacterium:* observation of discontinuities in amino acid identity values, a possible consequence of major extinction events, guides transfer of nine species to the genus *Epilithonimonas*, eleven species to the genus *Kaistella*, and three species to the genus *Halpernia* gen. nov., with description of *Kaistella daneshvariae* sp. nov. and *Epilithonimonas vandammei* sp. nov. derived from clinical specimens. Int J Syst Evol Microbiol. 2020;70:4432–50. PubMed https://doi.org/10.1099/ijsem.0.003935
